# Supplementary material for: Maternal cytokine profiles in second and early third trimester are not predictive of preterm birth
Source: PLoS One. 2024 Dec 19;19(12):e0311721. doi: 10.1371/journal.pone.0311721 (PMC11658620; doi:10.1371/journal.pone.0311721)
Supplement: S1 Table — Models are adjusted for subject specific intercepts and other maternal factors. Values are represented as slope between timepoints and 95% confidence intervals. Nonsignificant interactions between timepoint and group were removed from the models. (DOCX) [file pone.0311721.s001.docx]

**S1 Table. The difference between mean cytokine level between preterm birth groups and term births with group specific interactions**

|  |  | **Crude** | | |  | **Adjusted** | | | | | | |
| --- | --- | --- | --- | --- | --- | --- | --- | --- | --- | --- | --- | --- |
|  | **Timepoint** | **sPTL** | **PPROM** | **mPTB** | **Timepoint** | **sPTL** | **PPROM** | **mPTB** | **Time*sPTL** | **Time*PPROM** | **Time*mPTB** | **ICC** |
| **SAA** | **-1.30E+06** | -2.70E+05 | 1.70E+06 | -1.40E+06 | **-1.30E+06** | -3.90E+05 | 2.60E+06 | -1.20E+06 |  |  |  | 0.633 |
|  | **[-2.0e+06,-5.5e+05]** | [-2.1e+06, 1.6e+06] | [-6.3e+05, 4.0e+06] | [-3.6e+06, 8.9e+05] | **[-2.1e+06,-5.6e+05]** | [-2.3e+06, 1.5e+06] | [-1.1e+04, 5.2e+06] | [-3.5e+06, 1.1e+06] |  |  |  |  |
| **sICAM1** | **4.00E+04** | 6.00E+04 | 6474.353 | 1012.744 | **4.00E+04** | **7.50E+04** | -1.10E+04 | -1.20E+04 |  |  |  | 0.611 |
|  | **[1.5e+04, 6.6e+04]** | [-3.6e+03, 1.2e+05] | [-7.3e+04, 8.6e+04] | [-7.6e+04, 7.8e+04] | **[1.3e+04, 6.6e+04]** | **[1.1e+04, 1.4e+05]** | [-9.7e+04, 7.6e+04] | [-8.8e+04, 6.4e+04] |  |  |  |  |
| **Eotaxin** | **-107.489** | -9.133 | -2.553 | -7.018 | **-107.505** | -9.124 | 0.878 | -8.713 |  |  |  | 0.201 |
|  | **[-117.884,-97.093]** | [-25.265,6.999] | [-22.747,17.641] | [-26.424,12.388] | **[-118.431,-96.579]** | [-25.143,6.895] | [-20.973,22.729] | [-27.997,10.570] |  |  |  |  |
| **G-CSF** | -2.293 | 3.138 | -0.637 | 18.37 | **-8.329** | 0.464 | -5.831 | 7.017 | 5.021 | 7.147 | **20.923** | 0.852 |
|  | [-6.170,1.583] | [-12.388,18.664] | [-20.022,18.749] | [-0.212,36.953] | **[-15.029 ,-1.629]** | [-16.278 ,17.207] | [-28.49 ,16.828] | [-12.876 ,26.909] | [-4.517 ,14.559] | [-5.989 ,20.283] | **[9.325 ,32.52]** |  |
| **GM-CSF** | **-1.788** | -0.702 | 1.179 | 4.961 | **-1.721** | -0.964 | -2.863 | 4.982 |  |  |  | 0.929 |
|  | **[-3.375,-0.200]** | [-8.768,7.363] | [-8.889,11.248] | [-4.688,14.610] | **[-3.306,-0.136]** | [-9.008,7.081] | [-13.715,7.989] | [-4.596,14.560] |  |  |  |  |
| **IFNy** | -0.038 | 0.162 | -3.077 | -0.483 | -0.095 | -0.675 | -1.372 | 0.074 |  |  |  | 0.807 |
|  | [-1.940,1.865] | [-6.672,6.995] | [-11.611,5.456] | [-8.664,7.698] | [-2.158,1.967] | [-7.910,6.561] | [-11.148,8.403] | [-8.553,8.701] |  |  |  |  |
| **IL-1B** | -0.469 | 0.486 | 0.848 | 0.875 | -0.475 | 0.402 | 1.078 | 0.744 |  |  |  | 0.877 |
|  | [-1.039,0.100] | [-2.042,3.013] | [-2.307,4.003] | [-2.150,3.899] | [-1.092,0.141] | [-2.286,3.091] | [-2.550,4.707] | [-2.459,3.947] |  |  |  |  |
| **IL-1ra** | 2.489 | -3.592 | -0.629 | 7.36 | -5.349 | -10.139 | -14.338 | -4.071 | 10.743 | 12.507 | **18.239** | 0.691 |
|  | [-2.219,7.197] | [-16.238,9.054] | [-16.429,15.172] | [-7.796,22.516] | [-13.308 ,2.611] | [-23.993 ,3.715] | [-33.167 ,4.492] | [-20.52 ,12.378] | [-0.591 ,22.077] | [-3.07 ,28.083] | **[4.471 ,32.007]** |  |
| **IL-6** | 0.005 | 0.733 | -0.288 | 0.241 | -0.115 | 0.39 | -0.063 | 0.098 |  |  |  | 0.972 |
|  | [-0.422,0.433] | [-2.980,4.446] | [-4.922,4.346] | [-4.199,4.680] | [-0.530,0.299] | [-3.564,4.344] | [-5.391,5.266] | [-4.605,4.802] |  |  |  |  |
| **IL-8** | 11.918 | -5.965 | -6.197 | -1.182 | 11.064 | -6.817 | -11.37 | -1.984 |  |  |  | 1.08E-17 |
|  | [-0.143,23.978] | [-23.295,11.365] | [-27.898,15.503] | [-22.042,19.679] | [-1.660,23.788] | [-24.955,11.321] | [-36.122,13.383] | [-23.828,19.861] |  |  |  |  |
| **IL-10** | 0.209 | 3.009 | 1.73 | 0.871 | -0.659 | 2.993 | -0.236 | 0.017 | 0.528 | **3.369** | 1.008 | 0.948 |
|  | [-0.511,0.929] | [-1.076,7.094] | [-3.369,6.829] | [-4.015,5.757] | [-1.848 ,0.529] | [-1.141 ,7.127] | [-5.382 ,4.91] | [-4.903 ,4.937] | [-1.173 ,2.23] | **[1.24 ,5.498]** | [-1.05 ,3.065] |  |
| **MCP-1** | **36.594** | 13.47 | 5.01 | 4.856 | **34.411** | 15.958 | 3.732 | -6.405 |  |  |  | 0.829 |
|  | **[23.094,50.093]** | [-26.854,53.794] | [-45.474,55.493] | [-43.213,52.925] | **[21.938,46.885]** | [-26.268,58.184] | [-53.523,60.987] | [-56.606,43.795] |  |  |  |  |
| **TNFa** | 0.569 | 0.76 | -0.068 | 2.333 | -0.963 | -0.514 | -1.941 | 0.073 | 0.83 | 1.753 | **4.573** | 0.729 |
|  | [-0.466,1.604] | [-2.286,3.805] | [-3.873,3.736] | [-1.316,5.982] | [-2.718 ,0.793] | [-3.771 ,2.743] | [-6.364 ,2.482] | [-3.795 ,3.941] | [-1.67 ,3.329] | [-1.683 ,5.19] | **[1.536 ,7.61]** |  |
| **VEGF-A** | 0.14 | 4.182 | -1.835 | 13.085 | 0.149 | 3.541 | -1.366 | 13.067 |  |  |  | 0.926 |
|  | [-1.923,2.203] | [-8.011,16.376] | [-17.055,13.385] | [-1.500,27.670] | [-2.089,2.386] | [-9.459,16.541] | [-18.896,16.165] | [-2.407,28.541] |  |  |  |  |

The difference in mean cytokine level between preterm birth groups and term births between two timepoints adjusted for subject specific intercept, slope and other maternal factors. Interactions between group and timepoint of collection were reported if significantly different. Bold values indicate significant at alpha<0.05.
