# Supplementary material for: Maternal cytokine profiles in second and early third trimester are not predictive of preterm birth
Source: PLoS One. 2024 Dec 19;19(12):e0311721. doi: 10.1371/journal.pone.0311721 (PMC11658620; doi:10.1371/journal.pone.0311721)
Supplement: S1 File — Serum and plasma samples as part of a secondary analysis of a proof of principle study were analysed to validate the use of either biological fluid for measurement of cytokines. (PDF) [file pone.0311721.s002.pdf]

S1 File. Cytokine levels in matched serum and plasmas at the same mid-gestation timepoint.

To assess potential differences in cytokine level in serums and plasmas, 6 participants were selected from a proof of principle study who had provided matching serums and plasmas taken at the same timepoint, approved by the Conjoint Health Research Ethics Board at the University of Calgary (REB21-0712). These serum and plasma pairs were analysed for levels of eotaxin, G-CSF, GM-CSF, IFNy, IL-1B, IL-6, IL-8, IL-10, IL-1ra, MCP-1, MIP-1a, TNFa, VEGF-A using a multiplex inflammatory cytokine bead-based assay (EveTechnologies, Calgary, Alberta, Canada).

The differences observed in this study compared samples from T1 (plasma) and T2 (serum), and thus it is possible they are associated with biological-fluid specific difference, and not gestational age. Validation of serum and plasma samples from the same patient and timepoint showed no significant differences between most cytokines between serum and plasma (Fig S1). Of those with a significant difference, the pattern observed was opposite than that of the gestational age differences observed, suggesting the gestational age differences reported are likely an underestimate.

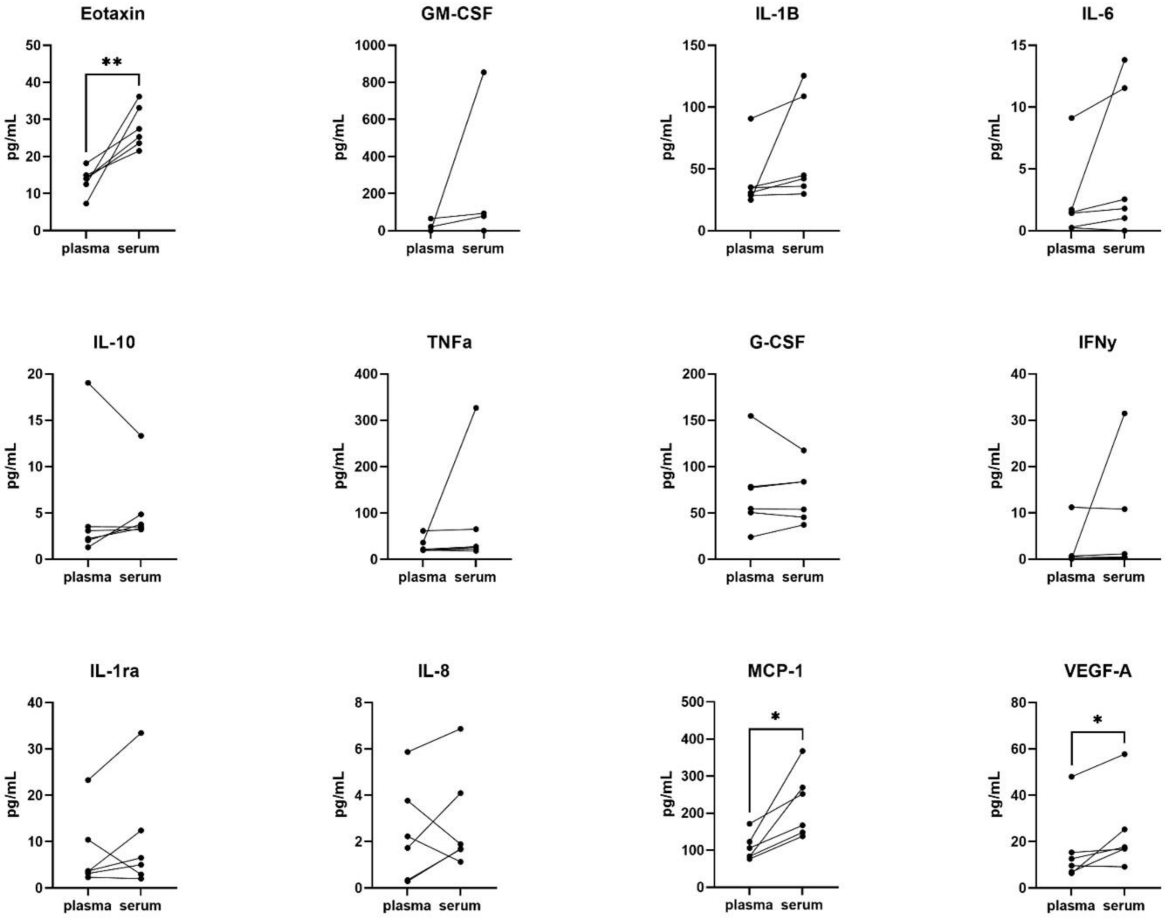

Fig S1. Eotaxin (mean difference 14.34±8.241pg/mL, p=0.0080), MCP-1 (mean difference 116.4±79.42pg/mL, p=0.0157), and VEGF-A (mean difference 7.450±7.066pg/mL, p=0.0493) levels were significantly higher in serum samples as compared to plasmas. No significant differences in levels of MIP-1a, GM-CSF, IL-1B, IL-6, IL-10, TNFa, G-CSF, IFNy, IL-1ra, or IL-8 were found with respect to biological fluid. Differences were analysed using Student's t-test. *p<0.05, **p<0.01. GM-CSF; granulocyte macrophage colony stimulated factor, IL-1B; interleukin 1 beta, IL-6; interleukin 6, IL-10; interleukin 10, TNFa; tumor necrosis factor alpha, G-CSF; granulocyte colony stimulating factor, IFNy; interferon gamma, IL-1ra; interleukin 1

receptor antagonist, IL-8; interleukin 8, MCP-1; monocyte chemoattractant protein 1, VEGF-A; vascular endothelial growth factor alpha.
