# Supplementary material for: Maternal cytokine profiles in second and early third trimester are not predictive of preterm birth
Source: PLoS One. 2024 Dec 19;19(12):e0311721. doi: 10.1371/journal.pone.0311721 (PMC11658620; doi:10.1371/journal.pone.0311721)
Supplement: S2 File — Crude and adjusted regression with subject-specific intercepts and slopes with time to delivery in weeks, as well as analysis to identify group specific interactions. (DOCX) [file pone.0311721.s003.docx]

**S2 Table 1: Linear mixed effects analysis of cytokine levels across preterm birth subtypes with time to delivery**

|  |  | **Crude RR** | | |  | **Adjusted RR** | | |
| --- | --- | --- | --- | --- | --- | --- | --- | --- |
|  | **Time to delivery (weeks)** | **sPTL** | **PPROM** | **mPTB** | **Time to delivery (weeks)** | **sPTL** | **PPROM** | **mPTB** |
| **SAA** | **-1.30E+05** | 3.80E+05 | 2.20E+06 | -7.50E+05 | **-1.30E+05** | 3.10E+05 | **3.00E+06** | -6.30E+05 |
|  | **[-2.0e+05,-5.3e+04]** | [-1.5e+06, 2.3e+06] | [-2.0e+05, 4.5e+06] | [-3.0e+06, 1.5e+06] | **[-2.1e+05,-5.2e+04]** | [-1.7e+06, 2.3e+06] | **[3.9e+05, 5.7e+06]** | [-3.0e+06, 1.7e+06] |
| **sICAM1** | **4259.399** | 3.30E+04 | -9.60E+03 | -2.60E+04 | **3945.999** | 4.30E+04 | -2.10E+04 | -3.30E+04 |
|  | **[1699.216,6819.582]** | [-2.7e+04, 9.4e+04] | [-8.5e+04, 6.5e+04] | [-9.9e+04, 4.7e+04] | **[1211.737,6680.260]** | [-1.7e+04, 1.0e+05] | [-1.0e+05, 6.1e+04] | [-1.1e+05, 3.9e+04] |
| **Eotaxin** | **-10.442** | **44.578** | **36.716** | **40.352** | **-10.43** | **45.971** | **37.04** | **36.072** |
|  | **[-11.474,-9.411]** | **[26.626,62.531]** | **[14.913,58.518]** | **[19.180,61.524]** | **[-11.515,-9.345]** | **[27.817,64.125]** | **[13.271,60.809]** | **[14.779,57.365]** |
| **G-CSF** | -0.287 | 3.974 | -0.7 | 13.483 | -0.271 | 3.576 | -3.2 | 10.996 |
|  | [-0.685,0.110] | [-11.284,19.232] | [-19.654,18.254] | [-4.806,31.773] | [-0.701,0.160] | [-12.309,19.461] | [-24.553,18.153] | [-7.953,29.944] |
| **GM-CSF** | **-0.196** | 0.322 | 1.892 | 5.884 | **-0.193** | 0.69 | -3.046 | 3.972 |
|  | **[-0.358,-0.034]** | [-7.769,8.414] | [-8.171,11.954] | [-3.773,15.540] | **[-0.358,-0.028]** | [-7.230,8.610] | [-13.716,7.624] | [-5.492,13.435] |
| **IFNy** | -0.002 | 0.197 | -3.152 | -0.518 | -0.005 | -0.629 | -1.411 | 0.093 |
|  | [-0.195,0.191] | [-6.732,7.125] | [-11.742,5.437] | [-8.776,7.739] | [-0.216,0.205] | [-7.966,6.707] | [-11.231,8.409] | [-8.605,8.791] |
| **IL-1B** | -0.054 | 0.77 | 1.047 | 1.117 | -0.056 | 0.703 | 1.262 | 0.978 |
|  | [-0.112,0.004] | [-1.771,3.312] | [-2.110,4.204] | [-1.914,4.148] | [-0.119,0.007] | [-2.002,3.408] | [-2.366,4.891] | [-2.231,4.188] |
| **IL-1ra** | 0.258 | -6.181 | -2.539 | 4.462 | 0.316 | -8.363 | -11.004 | 0.818 |
|  | [-0.220,0.737] | [-18.862,6.500] | [-18.209,13.130] | [-10.699,19.623] | [-0.182,0.815] | [-21.114,4.389] | [-28.076,6.068] | [-14.376,16.011] |
| **IL-6** | 0.002 | 0.723 | -0.295 | 0.227 | -0.011 | 0.447 | -0.029 | 0.14 |
|  | [-0.042,0.046] | [-2.998,4.444] | [-4.933,4.342] | [-4.218,4.673] | [-0.053,0.032] | [-3.513,4.408] | [-5.359,5.302] | [-4.568,4.847] |
| **IL-8** | **1.176** | -5.86 | -5.967 | 5.021 | 1.1 | -6.822 | -2.728 | 4.519 |
|  | **[0.119,2.233]** | [-19.232,7.511] | [-22.813,10.879] | [-11.935,21.977] | [-0.023,2.223] | [-20.628,6.984] | [-22.020,16.564] | [-12.937,21.974] |
| **IL-10** | 0.02 | 2.903 | 1.657 | 0.797 | -0.01 | 3.352 | 0.648 | 0.685 |
|  | [-0.053,0.094] | [-1.203,7.010] | [-3.454,6.767] | [-4.105,5.699] | [-0.075,0.054] | [-0.906,7.609] | [-5.077,6.373] | [-4.374,5.743] |
| **MCP-1** | **3.665** | -4.816 | -8.736 | -12.767 | **3.475** | -2.754 | -10.058 | -23.399 |
|  | **[2.286,5.044]** | [-44.408,34.775] | [-57.889,40.418] | [-60.647,35.113] | **[2.196,4.754]** | [-44.386,38.879] | [-66.194,46.077] | [-73.399,26.600] |
| **TNFa** | 0.035 | 0.563 | -0.276 | 2.054 | 0.011 | -0.19 | -1.181 | 1.997 |
|  | [-0.070,0.140] | [-2.538,3.663] | [-4.109,3.557] | [-1.639,5.746] | [-0.099,0.121] | [-3.306,2.926] | [-5.338,2.976] | [-1.692,5.685] |
| **VEGF-A** | 0.015 | 4.101 | -1.892 | 13.049 | 0.017 | 3.45 | -1.419 | 13.03 |
|  | [-0.195,0.226] | [-8.145,16.348] | [-17.135,13.351] | [-1.573,27.670] | [-0.212,0.246] | [-9.612,16.511] | [-18.970,16.133] | [-2.483,28.543] |

The difference in mean cytokine level between preterm birth groups and term births at baseline and the rate of change in the mean cytokine level until delivery, adjusted for subject specific intercept, slope and other maternal factors. Values are reported as risk ratios [95% confidence interval] for each group as primary exposure variables, and timepoint of collection (time) as a fixed effect parameter. Cytokine levels for SAA and sICAM-1 were transformed by centering around the minimum value prior to analysis. Bold values indicate significantly non-zero.


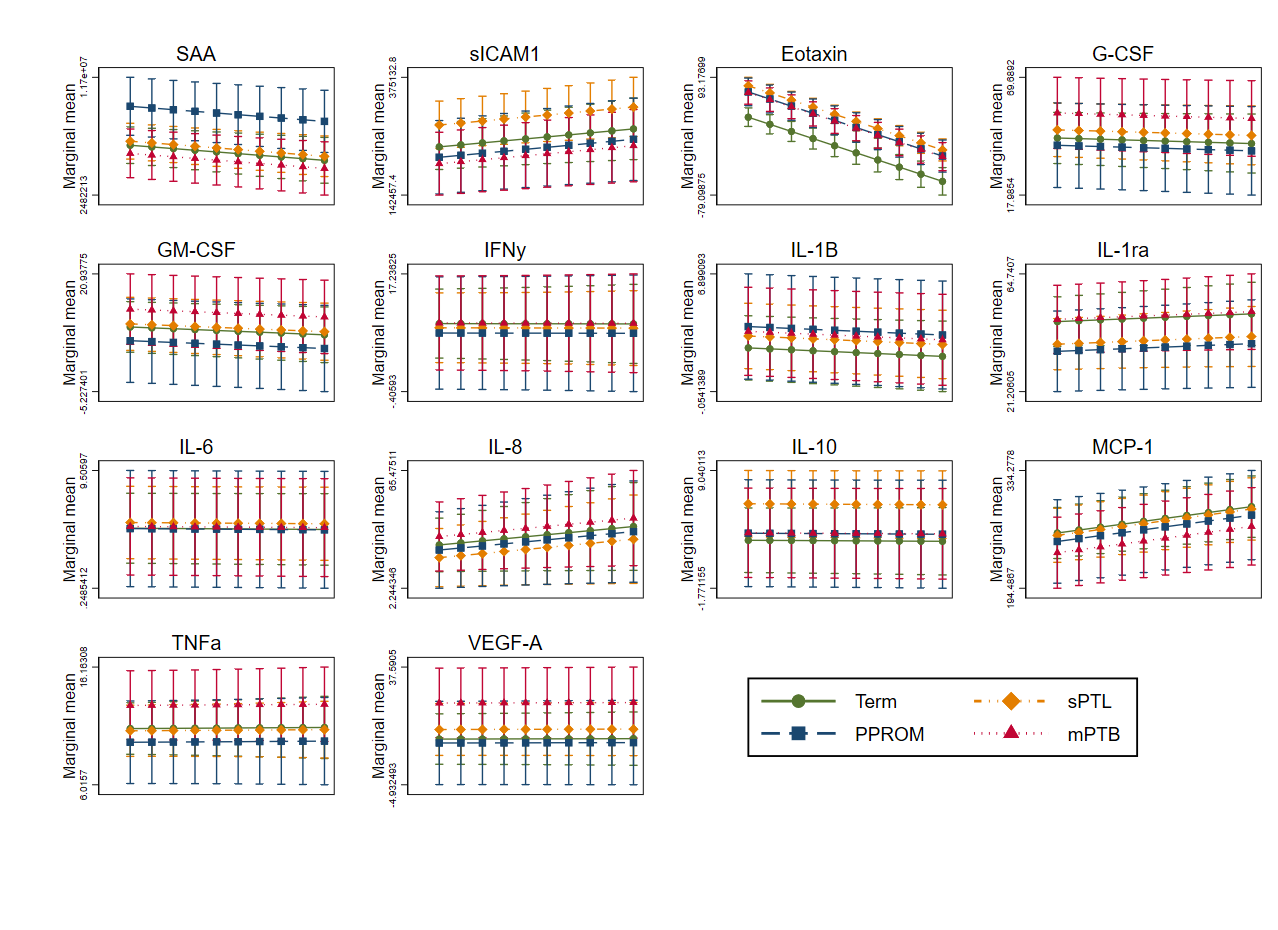


**S2 Figure 1. The difference in mean cytokine level between preterm brith groups and term births at baseline and the rate of change in the mean cytokine level until delivery.** Figures were produced using the marginal model error variance structure at two timepoints in pregnancy, showing marginal mean levels of cytokine (y-axis), across two timepoints in pregnancy (x-axis).

**S2 Table 2: The difference between mean cytokine level between preterm birth groups and term births with group specific interactions**

|  |  | **Crude RR** | | |  | **Adjusted RR** | | |  | **Interaction** |  |  |
| --- | --- | --- | --- | --- | --- | --- | --- | --- | --- | --- | --- | --- |
|  | **Time to delivery (weeks)** | **sPTL** | **PPROM** | **mPTB** | **Time to delivery (weeks)** | **sPTL** | **PPROM** | **mPTB** | **Time*sPTL** | **Time*PPROM** | **Time*mPTB** | **ICC** |
| **SAA** | **-1.30E+05** | 3.80E+05 | 2.20E+06 | -7.50E+05 | **-1.30E+05** | 3.10E+05 | **3.00E+06** | -6.30E+05 |  |  |  | 0.639686 |
|  | **[-2.0e+05,-5.3e+04]** | [-1.5e+06, 2.3e+06] | [-2.0e+05, 4.5e+06] | [-3.0e+06, 1.5e+06] | **[-2.1e+05,-5.2e+04]** | [-1.7e+06, 2.3e+06] | **[3.9e+05, 5.7e+06]** | [-3.0e+06, 1.7e+06] |  |  |  |  |
| **sICAM1** | **4259.399** | 3.30E+04 | -9.60E+03 | -2.60E+04 | **3945.999** | 4.30E+04 | -2.10E+04 | -3.30E+04 |  |  |  | 0.605588 |
|  | **[1699.216,6819.582]** | [-2.7e+04, 9.4e+04] | [-8.5e+04, 6.5e+04] | [-9.9e+04, 4.7e+04] | **[1211.737,6680.260]** | [-1.7e+04, 1.0e+05] | [-1.0e+05, 6.1e+04] | [-1.1e+05, 3.9e+04] |  |  |  |  |
| **Eotaxin** | **-10.442** | **44.578** | **36.716** | **40.352** | **-10.43** | **45.971** | **37.04** | **36.072** |  |  |  | 0.271269 |
|  | **[-11.474,-9.411]** | **[26.626,62.531]** | **[14.913,58.518]** | **[19.180,61.524]** | **[-11.515,-9.345]** | **[27.817,64.125]** | **[13.271,60.809]** | **[14.779,57.365]** |  |  |  |  |
| **G-CSF** | -0.287 | 3.974 | -0.7 | 13.483 | **-0.917** | 1.902 | -7.032 | -7.266 | 0.464 | 0.722 | **2.332** | 0.862392 |
|  | [-0.685,0.110] | [-11.284,19.232] | [-19.654,18.254] | [-4.806,31.773] | **[-1.586 ,-0.248]** | [-16.848 ,20.652] | [-32.459 ,18.396] | [-30.232 ,15.701] | [-0.464 ,1.392] | [-0.57 ,2.013] | **[1.124 ,3.539]** |  |
| **GM-CSF** | **-0.196** | 0.322 | 1.892 | 5.884 | **-0.193** | 0.69 | -3.046 | 3.972 |  |  |  | 0.950671 |
|  | **[-0.358,-0.034]** | [-7.769,8.414] | [-8.171,11.954] | [-3.773,15.540] | **[-0.358,-0.028]** | [-7.230,8.610] | [-13.716,7.624] | [-5.492,13.435] |  |  |  |  |
| **IFNy** | -0.002 | 0.197 | -3.152 | -0.518 | -0.005 | -0.629 | -1.411 | 0.093 |  |  |  | 0.806381 |
|  | [-0.195,0.191] | [-6.732,7.125] | [-11.742,5.437] | [-8.776,7.739] | [-0.216,0.205] | [-7.966,6.707] | [-11.231,8.409] | [-8.605,8.791] |  |  |  |  |
| **IL-1B** | -0.054 | 0.77 | 1.047 | 1.117 | -0.056 | 0.703 | 1.262 | 0.978 |  |  |  | 0.86973 |
|  | [-0.112,0.004] | [-1.771,3.312] | [-2.110,4.204] | [-1.914,4.148] | [-0.119,0.007] | [-2.002,3.408] | [-2.366,4.891] | [-2.231,4.188] |  |  |  |  |
| **IL-1ra** | 0.258 | -6.181 | -2.539 | 4.462 | -0.394 | -14.535 | -18.812 | -12.538 | 0.923 | 1.103 | **1.603** | 0.692686 |
|  | [-0.220,0.737] | [-18.862,6.500] | [-18.209,13.130] | [-10.699,19.623] | [-1.215 ,0.426] | [-31.749 ,2.679] | [-42.305 ,4.682] | [-34.07 ,8.993] | [-0.214 ,2.059] | [-0.476 ,2.682] | **[0.13 ,3.076]** |  |
| **IL-6** | 0.002 | 0.723 | -0.295 | 0.227 | -0.011 | 0.447 | -0.029 | 0.14 |  |  |  | 0.971727 |
|  | [-0.042,0.046] | [-2.998,4.444] | [-4.933,4.342] | [-4.218,4.673] | [-0.053,0.032] | [-3.513,4.408] | [-5.359,5.302] | [-4.568,4.847] |  |  |  |  |
| **IL-8** | **1.176** | -5.86 | -5.967 | 5.021 | 1.1 | -6.822 | -2.728 | 4.519 |  |  |  | 2.85E-13 |
|  | **[0.119,2.233]** | [-19.232,7.511] | [-22.813,10.879] | [-11.935,21.977] | [-0.023,2.223] | [-20.628,6.984] | [-22.020,16.564] | [-12.937,21.974] |  |  |  |  |
| **IL-10** | 0.02 | 2.903 | 1.657 | 0.797 | -0.074 | 3.227 | -1.951 | -0.24 | 0.037 | **0.264** | 0.105 | 0.924796 |
|  | [-0.053,0.094] | [-1.203,7.010] | [-3.454,6.767] | [-4.105,5.699] | [-0.18 ,0.033] | [-1.269 ,7.723] | [-8.024 ,4.123] | [-5.658 ,5.179] | [-0.11 ,0.184] | **[0.059 ,0.469]** | [-0.087 ,0.297] |  |
| **MCP-1** | **3.665** | -4.816 | -8.736 | -12.767 | **3.475** | -2.754 | -10.058 | -23.399 |  |  |  | 0.884067 |
|  | **[2.286,5.044]** | [-44.408,34.775] | [-57.889,40.418] | [-60.647,35.113] | **[2.196,4.754]** | [-44.386,38.879] | [-66.194,46.077] | [-73.399,26.600] |  |  |  |  |
| **TNFa** | 0.035 | 0.563 | -0.276 | 2.054 | 0.011 | -0.19 | -1.181 | 1.997 | 0.83 | 1.753 | **4.573** | 0.925184 |
|  | [-0.070,0.140] | [-2.538,3.663] | [-4.109,3.557] | [-1.639,5.746] | [-0.099,0.121] | [-3.306,2.926] | [-5.338,2.976] | [-1.692,5.685] | [-1.67 ,3.329] | [-1.683 ,5.19] | **[1.536 ,7.61]** |  |
| **VEGF-A** | 0.015 | 4.101 | -1.892 | 13.049 | 0.017 | 3.45 | -1.419 | 13.03 |  |  |  | 0.925184 |
|  | [-0.195,0.226] | [-8.145,16.348] | [-17.135,13.351] | [-1.573,27.670] | [-0.212,0.246] | [-9.612,16.511] | [-18.970,16.133] | [-2.483,28.543] |  |  |  |  |

The difference in mean cytokine level between preterm birth groups and term births at baseline and the rate of change in the mean cytokine level until delivery adjusted for subject specific intercept, slope and other maternal factors. Interactions between group and time to delivery were reported if significantly different. Bold values indicate significant at alpha<0.05.


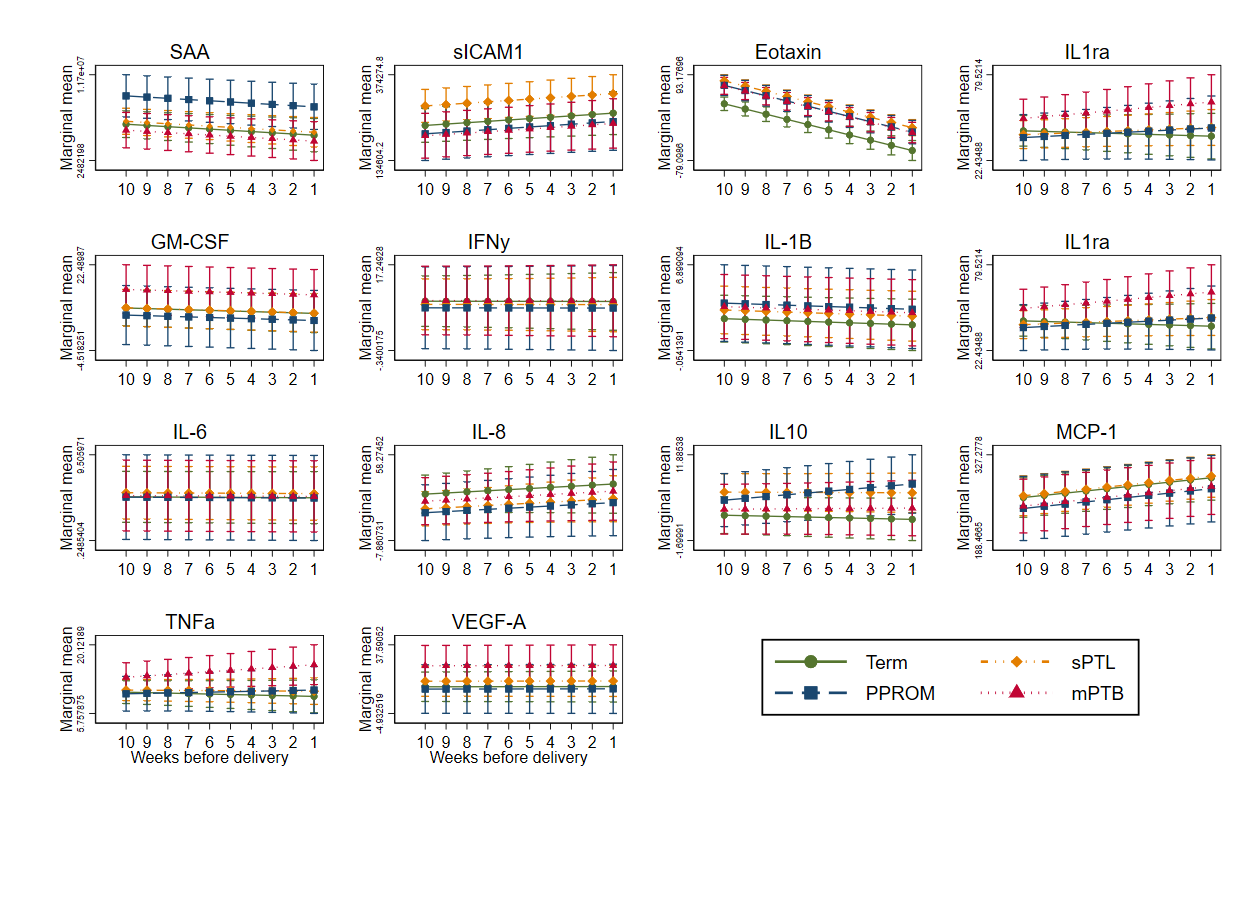


**S2 Figure 2. Rate of change in mean cytokine level with time to delivery across preterm and term groups with group specific interactions.** Models were adjusted for subject specific intercepts and other maternal factors. Non-significant interaction terms between groups and time were removed. Figures were produced using the marginal model error variance structure at two timepoints in pregnancy.
